# Hydroxychloroquine’s diverse targets: a new frontier in precision medicine

**DOI:** 10.3389/fimmu.2025.1655573

**Published:** 2025-10-08

**Authors:** Bin Du, Leqi Li, Jingjing Li, Yiping Liu, Pu Wang

**Affiliations:** ^1^ Shanxi Key Laboratory of Aging Mechanism Research and Translational Applications, Changzhi Medical College, Changzhi, China; ^2^ School of Basic Medical Sciences, Changzhi Medical College, Changzhi, China

**Keywords:** hydroxychloroquine, autophagy, mitochondrial damage, nucleic acid targeting, membrane structure remodeling, zinc homeostasis, clinical application

## 1 Introduction

Hydroxychloroquine (HCQ), an economically accessible pharmaceutical with multifaceted therapeutic effects, is utilized in the management of both autoimmune and infectious diseases. Within the realm of rheumatology, HCQ is a fundamental treatment for systemic lupus erythematosus (SLE) (1), where it functions by inhibiting Toll-like receptor (TLR7/9)-mediated nucleic acid recognition, elevation of lysosomal pH and the reduction of type I interferon secretion  (2). In the context of rheumatoid arthritis (RA), HCQ plays a role in the suppression of B-cell activation and the production of proinflammatory cytokines, such as interleukin-6 (IL-6) and tumor necrosis factor-alpha (TNF-α), with its combination with methotrexate leading to significant improvements in disease activity (3). Furthermore, HCQ has been shown to mitigate the risk of thrombosis in antiphospholipid syndrome by inhibiting the formation of neutrophil extracellular traps (NETs)  (4). Early studies on COVID-19 suggested that HCQ might interfere with viral envelope protein glycosylation and modulate cytokine storms, indicating potential therapeutic benefits  (5).

The clinical application of HCQ is constrained by its complex dose-dependent toxicity profile. Prolonged use is associated with irreversible photoreceptor damage resulting from accumulation in retinal pigment epithelial cells, which carries an approximate 20% risk of cumulative blindness over 20 years. This condition initially manifests as “bull’s-eye” macular pigmentation (6). In the cardiovascular system, administration of high doses exceeding 5 mg/kg can lead to QT interval prolongation, myocardial fibrosis, and conduction blockades (7). A study involving 127 patients revealed that 39.4% of those undergoing HCQ treatment experienced cardiac conduction dysfunction (8). Cutaneous toxicity, with an incidence rate of 10% to 20%, encompasses mucosal and acral hyperpigmentation, drug eruptions, and pruritus (9). Muscular toxicity is characterized by proximal myasthenia and elevated creatine kinase levels, which may advance to respiratory muscle paralysis (10). Gastrointestinal reactions, such as nausea and diarrhea, are associated with dysbiosis, while patients with hepatic or renal insufficiency are at risk of drug accumulation (11). These adverse effects underscore the importance of continuous monitoring during treatment.

Understanding the mechanisms of HCQ is essential for addressing clinical challenges. HCQ has been shown to reduce cardiovascular events in patients with rheumatoid arthritis (RA)  (12, 13) and to exhibit therapeutic effects in a murine model of myocarditis  (14). However, it can also induce arrhythmias in some COVID-19 patients and cause cardiotoxicity with prolonged use  (11, 15).

Despite extensive research, comprehensive reviews focusing on HCQ’s direct targets and mechanisms are limited. This review seeks to systematically elucidate HCQ’s interactions with nucleic acids, proteins, and lipids, thereby uncovering the fundamental mechanisms responsible for its therapeutic benefits and adverse effects. Such insights will aid in developing personalized treatment strategies based on biomarkers (e.g., circulating tumor DNA levels, lysosomal enzyme activity), thereby transforming HCQ’s “double-edged sword” effect into an advantage for precision medicine.

## 2 Targets and mechanisms of hydroxychloroquine

### 2.1 Nucleic acid targeting mechanisms

#### 2.1.1 DNA binding properties

The binding of HCQ to DNA was initially identified using optical tweezers, which revealed the formation of a complex with DNA (16). HCQ interacts with double-stranded DNA (dsDNA) in a manner dependent on both concentration and sequence. At low concentrations, HCQ associates with the minor groove of DNA through electrostatic interactions. In contrast, at higher concentrations, intercalation occurs, as confirmed by single-molecule force spectroscopy and gel electrophoresis (17). Thermodynamic analyses indicate that this binding process is spontaneous and predominantly driven by electrostatic interactions, with binding constants decreasing as temperature increases (16, 18).

HCQ demonstrates sequence-specific selectivity in its interaction with DNA, exhibiting a notably higher binding affinity for guanine-cytosine (GC)-rich regions compared to adenine-thymine (AT)-rich regions (19). In GC-rich DNA, HCQ predominantly binds through the major groove with intercalation, whereas in AT-rich DNA, binding occurs primarily via the minor groove. The presence of salt ions, such as Mg²^+^ and Na^+^, influences this sequence-specific binding; Mg²^+^ ions enhance HCQ binding to AT-rich DNA but inhibit its binding to GC-rich DNA, a phenomenon attributed to variations in ion hydration and charge distribution within the DNA grooves (20). In patients with SLE, plasma concentrations of HCQ within the therapeutic range (0.5–1.0 μg/mL) show a positive correlation with the binding rates to cell-free DNA (cfDNA). The binding of HCQ to cfDNA significantly impedes the interaction of DNA with Toll-like receptor 9 (TLR9), resulting in a 40% reduction in the cfDNA-TLR9 complex (21). Furthermore, HCQ obscures CpG motifs in nucleosomal DNA, thereby preventing the activation of TLR9 in plasmacytoid dendritic cells (pDCs) and subsequently reducing the secretion of type I interferons (21, 22). In tumor-related research, hydroxychloroquine has also been reported to promote tumor metastasis through a dual mechanism of inhibiting the formation of NETs and blocking the TLR4/9-COX2 signaling pathway activated by extracellular trap DNA (23, 24). In diseases associated with altered immune microenvironment, hydroxychloroquine has shows promising therapeutic potentiall.

#### 2.1.2 Regulation of G4 DNA

Recent research indicates that HCQ influences the stability of intracellular G-quadruplexes (G4) (25). HCQ promotes the formation of G4 structures within telomeric G-rich sequences, where the number of flanking nucleotides plays a crucial role in determining G4 folding and stability. Specifically, single-side flanking nucleotides exert minimal impact, whereas the presence of multiple flanking nucleotides on both sides markedly reduces the propensity for G4 folding and stability (25). Regarding topological selectivity, HCQ exhibits a preference for binding to human telomere hybrid-type G4 structures (26), effectively stabilizing telomeric G4 and preventing its transition to dsDNA. In typical cellular environments, which are predominantly characterized by the presence of potassium ions, HCQ demonstrates a negligible effect (27). The mechanism of HCQ binding to G4 involves π-π stacking, C-H˙˙˙π interactions, and hydrogen bonding between positively charged side chains and guanine quartets (25).

The DNA of malaria parasites is rich in G-quadruplex sequences, and therapies targeting these G4 structures have demonstrated promising antimalarial effects (28, 29). Considering HCQ’s ability to bind to G4 structures, it may offer a low-toxicity therapeutic option for malaria patients who also suffer from autoimmune diseases. Furthermore, this property of HCQ could inspire the development of next-generation antimalarial ligands aimed at overcoming drug resistance (30).

#### 2.1.3 RNA interaction network

Research on the binding interactions between HCQ and RNA emerged in response to the COVID-19 pandemic (31), while Early hypotheses and preliminary studies initially suggested a potential therapeutic benefit of HCQ for COVID-19, subsequent, more robust clinical trials and analyses have not supported its efficacy in treating COVID-19. Thermodynamic analyses indicate that HCQ associates with RNA grooves through an entropy-driven mechanism, with a preference for uridine/cytidine-rich regions (31). These results imply that HCQ could potentially inhibit Toll-like receptor 8 (TLR8) recognition of RNA from Plasmodium falciparum-infected erythrocytes or NETs in SLE patients, thereby diminishing interferon-gamma (IFN-γ) secretion and mitigating inflammatory responses (32, 33).

HCQ specifically interacts with the stem-loop II motif (s2m) of SARS-CoV-2 RNA, promoting the dimerization of this element, which subsequently disrupts viral envelope protein maturation and RNA proofreading (34, 35). Additionally, HCQ binds to the antiterminator RNA of T-box riboswitches, thereby inhibiting transcriptional antitermination (36, 37). The RNA of the malaria parasite is characterized by a pronounced A/T bias and an abundance of hairpins and multi-stem loops, which are essential for parasite infection (38–40). A comparative analysis of HCQ-bound T-box RNA and the structural attributes of parasite RNA reveals notable similarities, indicating that HCQ may directly associate with parasite RNA. This interaction could disrupt RNA metabolism, interfere with the temperature-responsive conformational changes of 18S rRNA, and ultimately inhibit parasite development within red blood cells (38).

RNA G-quadruplex structures are also present in A/U-rich sequences of Plasmodium falciparum, particularly within the RNA of virulence-associated genes, such as the var gene family, and in telomeric regions (30, 39, 41). These G4 structures frequently exhibit non-canonical forms, such as bulged or double quadruplexes, and overlap with regions coding for low-complexity peptides, playing a role in the regulation of antigenic variation. Considering the established ability of HCQ to bind DNA G4 structures, it is plausible that HCQ may also inhibit RNA G4s, potentially contributing to its pharmacological effects.

### 2.2 Protein interaction landscape

Historically, the disruption of autophagic flux was regarded as the primary mechanism by which chloroquine influences cellular function (42). However, experimental studies utilizing zebrafish and tumor models have demonstrated that HCQ exhibits significant antitumor activity that is independent of autophagy (43). Network pharmacology analyses have identified potential targets of HCQ across various diseases, including cyclin-dependent kinase 2 (CDK2), matrix metalloproteinase 2 (MMP2), and smoothened (SMO), among others (44–46). Furthermore, molecular docking studies and experimental data indicate that HCQ directly interacts with proteins such as palmitoyl-protein thioesterase 1 (PPT1) (47), nucleocapsid phosphoprotein (48), angiotensin-converting enzyme 2 (ACE2) (49), and the α7 nicotinic acetylcholine receptor (α7 nAChR) (50).

A recent investigation employing Thermal Proteome Profiling technology has identified that HCQ may directly interact with several proteins, including NQO2, GSR, NAMPT, KIF11, SEC23A, PUF60, PCM1, and NONO, among others (51). Notably, proteins such as SEC23A, PCM1, and ARFGAP1 have been implicated in the autophagic process (52–54), thereby enhancing our comprehension of HCQ’s role. However, the precise molecular mechanism by which HCQ impedes autophagy, specifically through the reduction of autophagosome-lysosome fusion, remains elusive (42). This ambiguity may be attributed to brief cell treatment durations or inherent technical limitations (51).

HCQ has been identified as an antithrombotic agent in the treatment of antiphospholipid syndrome and SLE (55–57), with a favorable safety profile (58). Mechanistically, HCQ inhibits the formation of antiphospholipid (aPL) IgG-beta2-glycoprotein I (beta2GPI) complexes and reduces protein binding to phospholipid bilayers (59, 60). Additionally, HCQ counteracts the effects of antiphospholipid antibodies on annexin A5 (AnxA5), facilitates the restoration of AnxA5 binding to phospholipid bilayers, and reinstates AnxA5’s anticoagulant properties (61). These actions elucidate HCQ’s mechanism in the management of childhood chronic immune thrombocytopenia (62).

### 2.3 Disruption of pH homeostasis

Chloroquine (CQ) and HCQ, as dibasic weak alkaline drugs, exist in both protonated and unprotonated forms. The unprotonated forms of CQ and HCQ are capable of freely traversing cell membranes and diffusing into acidic organelles, such as lysosomes and Golgi lumens, where they neutralize the acidic pH (63). Numerous studies utilizing probes have documented the impact of hydroxychloroquine and chloroquine on lysosomal pH (64, 65). Notably, treatment with 200 μM chloroquine for one hour elevates the intracellular lysosomal pH from 5.3 ± 0.1 to 6.6 ± 0.1, without altering the overall cytoplasmic pH (66). A reduction in lysosomal acidity can trigger a cascade of effects, including the inactivation of acidic hydrolases within lysosomes, accumulation of metabolic waste products, decreased autophagic efficiency, and other related complications (67). Nonetheless, it remains uncertain whether the elevation in lysosomal pH directly influences autophagosome-lysosome fusion. The identification of direct binding targets of HCQ, such as SEC23A, PCM1, and ARFGAP1, implies that HCQ may inhibit autophagosome-lysosome fusion through more direct mechanisms (51).

### 2.4 Membrane structure remodeling

#### 2.4.1 Lysosomes

Lysosomal membranes possess a distinct composition characterized by the presence of a unique anionic lipid, bis(monoacylglycero)phosphate (BMP), which serves as a lysosomal marker not found in other intracellular membranes (68). Research indicates that HCQ interferes with the hydrogen bond network of water molecules by directly integrating into negatively charged phospholipid membranes, akin to those of lysosomal membranes, rather than amphoteric phospholipids. This integration results in water structure disorder or induces “gauche defects” in the hydrocarbon chains, leading to a disordered arrangement of membrane lipids and alterations in the membrane’s physical properties and composition (69). Such disruptions may influence the localization and activity of critical fusion proteins, including SNARE protein complexes and Rab GTPases, on the membrane. Consequently, this interference can impede the docking and fusion of autophagosome-lysosome membranes, thereby obstructing autophagosome-lysosome fusion and ultimately diminishing the efficiency of autophagic degradation (70).

#### 2.4.2 Mitochondria

Numerous studies have demonstrated that the use of HCQ significantly elevates intracellular reactive oxygen species (ROS) levels (71). As the primary organelle responsible for ROS production, mitochondria in myocardial cells undergo rapid alterations within 60 minutes of HCQ treatment at a concentration of 100 μM. These alterations include the collapse of mitochondrial membrane potential, mitochondrial swelling, and the release of cytochrome C (72). In contrast, the same concentration of HCQ takes a longer duration to substantially inhibit autophagy (42). Cardiac cell mitochondrial membranes contain approximately 15–30% anionic lipids, representing the highest content among all membrane-bound organelles (73–75). Consequently, upon cellular entry, HCQ preferentially targets mitochondrial membranes due to their abundant anionic lipid content, resulting in rapid mitochondrial membrane damage, disruption of the respiratory chain, and increased ROS production (76).

Furthermore, related research indicates that HCQ can directly interact with proteins such as glutathione reductase (GSR) (51), suggesting its potential to interfere with glutathione (GSH) production (77), and consequently promote an increase in ROS. Additional studies reveal that HCQ binds to the hydrophobic regions of membrane phospholipids, resulting in the neutralization of phosphate groups and displacement of calcium ions, thereby causing mitochondrial damage (78). Animal studies have demonstrated that HCQ treatment leads to an increase in phospholipids and a reduction in cholesterol within mitochondrial membranes (79), which implies decreased membrane fluidity and functional impairment. Ultrastructural analysis of cardiac tissue from a patient with HCQ-induced acute left ventricular failure corroborated mitochondrial damage, exhibiting membrane-bound concentric lamellar structures, aggregates of curvilinear bodies (In fact, this represents a manifestation of structural abnormalities in intracellular membrane structures), and variations in morphology and size, accompanied by damage to the mitochondrial cristae (80). These findings elucidate the pharmacological mechanisms underlying hydroxychloroquine-induced cardiotoxicity. In future clinical applications, the co-administration of mitochondrial-protective agents, such as melatonin, edaravone, tadalafil (81), and coenzyme Q10, may be considered to mitigate.

## 3 Strategies to reduce HCQ side effects and enhance efficacy

### 3.1 Liposomal carriers

Liposomes have the capability to target glioma, melanoma cells, or activated hepatic stellate cells through peptide modifications, such as the Angiopep-2 peptide (82) and the R8-dGR peptide (83), or through the application of double-membrane hybrid nano-bionic technology (84). The use of pH-sensitive release mechanisms, exemplified by Chol-HCQ liposomes (85), or copper complexation technology (86), facilitates targeted drug release within tumor microenvironments or lysosomes, achieving approximately an 850-fold increase in efficacy compared to free HCQ. The co-loading of liposomes with chemotherapeutic agents like paclitaxel (87) or photosensitizers (82) results in a synergistic inhibition of autophagy or an enhancement of phototherapy effects. Additionally, a novel lipid nanoparticle formulation that co-delivers HCQ and TNF-α-targeting siRNA (siTNF-α) has been developed for the treatment of rheumatoid arthritis (88). Empirical studies have demonstrated that Chol-HCQ liposomes effectively inhibit pulmonary inflammation and mitigate bleomycin-induced pulmonary fibrosis, with the safety of systemic administration confirmed 28 days following intravenous injection (85). While this study did not include data specifically addressing retinal or cardiac toxicity, it is hypothesized that liposomal encapsulation could mitigate the potential toxicity of HCQ to organs such as the retina and heart. This hypothesis is based on the safety verification and the principle that targeted drug delivery minimizes systemic exposure. Furthermore, liposomal delivery of HCQ has the potential to accumulate in lysosomes, thereby enhancing the efficiency of autophagy inhibition (86), and to achieve precise delivery and efficacy enhancement by modulating the activation of immune cells within the tumor microenvironment (87).

### 3.2 Chiral hydroxychloroquine

HCQ possesses a chiral center, resulting in two optical isomers: (-)-(R)-hydroxychloroquine and (+)-(S)-hydroxychloroquine. These chiral isomers exhibit distinct pharmacokinetic profiles, target binding affinities, and pharmacological effects. Notably, the R (-) and S (+) enantiomers of HCQ demonstrate significant differences in cardiac electrophysiology and calcium ion regulation. Specifically, R (-) HCQ markedly depolarizes the resting membrane potential of rabbit Purkinje fibers and induces spontaneous electrical activity, whereas S (+) HCQ predominantly prolongs the action potential duration, exhibiting 2–4-fold enantioselective inhibition of hERG and potassium channels (89). In human induced pluripotent stem cell-derived cardiomyocytes, R (-) HCQ inhibits Ca²^+^ oscillations with significantly greater potency than both S (+) HCQ and the racemic mixture (90). Furthermore, *in vivo* studies in rabbits indicate that R (-) HCQ achieves significantly higher concentrations in whole blood compared to S (+) HCQ, potentially due to its enhanced binding affinity for blood cell components (91).

Studies on anti-SARS-CoV-2 agents indicate that the R (-) enantiomer of HCQ exhibits superior *in vitro* antiviral activity and reduced *in vivo* toxicity compared to the S (+) enantiomer (92). Conversely, the S (+) enantiomer demonstrates a significantly enhanced binding affinity to the ACE2 receptor relative to both the R (-) enantiomer and the racemic mixture (93). Regarding ocular distribution, HCQ enantiomers display reversible enantioselective binding in the non-pigmented ocular tissues of rabbits (94). In patients with rheumatoid arthritis, plasma concentrations of R (-) HCQ are generally higher than those of S (+) HCQ, with markedly different clearance rates observed between the two enantiomers (95).

## 4 Clinical hydroxychloroquine usage strategy, based on the multi-targets

### 4.1 Treatment of autoimmune diseases

Beyond its established applications in rheumatoid arthritis and systemic lupus erythematosus, HCQ holds particular value as an adjunctive treatment in obstetrics, primarily due to its capacity to inhibit phospholipid antibody complex formation. This capability enables it to effectively prevent or manage conditions such as placental insufficiency, recurrent miscarriage, preterm birth, fetal growth restriction, and preeclampsia associated with antiphospholipid syndrome (57, 60, 96–99). In the cardiovascular context, HCQ is associated with a reduced risk of cardiovascular events in patients with hypertension, diabetes, or rheumatoid arthritis, mediated through anti-phospholipid antibody mechanisms (100–102), and it also confers neuroprotective benefits post-stroke (103). Additionally, HCQ is employed in the management of various other autoimmune-related conditions, including myositis, systemic sarcoidosis, cutaneous sarcoidosis, and granulomatous skin eruptions (104–108).

Cardiac and retinal toxicities from HCQ are linked to mitochondrial damage and elevated ROS (109). Importantly, HCQ significantly inhibits the activity of zinc-dependent antioxidant enzymes (110), and the maintenance of zinc ion homeostasis is essential for preserving cardiac function (111). This implies that dysregulated zinc metabolism may represent a fundamental mechanism underlying HCQ toxicity. Recent mechanistic studies further substantiate this hypothesis: Zhang et al. reported that HCQ directly interacts with the zinc transporter SLC30A7 and glutathione disulfide reductase (GSR), thereby disrupting intracellular zinc metabolism and antioxidant equilibrium (51). Studies show zinc supplementation counteracts HCQ-induced zinc reduction in Drosophila larvae (112) and prevents cardiac damage in rats (113). These findings imply that aberrant zinc transport caused by HCQ is a significant contributor to its side effects. Additionally, HCQ-induced neurological toxicity exhibits ultrastructural similarities to its cardiotoxicity (114). Consequently, we advocate for appropriate zinc supplementation in patients undergoing long-term conventional or short-term high-dose HCQ treatments to reduce the risk of cardiac, retinal, and neurological toxicities.

### 4.2 Treatment of infectious diseases

In the realm of antimalarial therapy, the structural modification of HCQ may represent a promising avenue for future research. Notably, the synthesis of zinc-hydroxychloroquine complexes (115, 116) by altering the conformation of HCQ and its distribution within plasmodia, shows potential in combating drug resistance arising from mutations in the Pfcrt and Pfmdr1 genes, which interfere with HCQ transport. Moreover, post-malaria infection, the activation of the SCC/PSAC channel on the erythrocyte membrane occurs, with zinc ions serving as natural inhibitors of this channel (117). Epidemiological studies have further suggested a correlation between zinc deficiency and an elevated risk of malaria infection (118). Building upon existing research on the role of metal ions in anti-infective therapy (119), we propose that the development of zinc-HCQ complexes could facilitate targeted action against infected erythrocytes and plasmodia, potentially reversing drug resistance in malaria parasites and offering a novel strategy for antimalarial treatment. Additionally, several studies have demonstrated the efficacy of HCQ in the treatment of Leishmania infection (120, 121).

### 4.3 Cancer therapy

As an inhibitor of autophagy, the effectiveness of HCQ in cancer therapy is significantly influenced by the mechanistic context of combination treatment regimens. Empirical studies have demonstrated that HCQ does not enhance the efficacy of DNA-targeting agents—such as temozolomide, gemcitabine in conjunction with nab-paclitaxel, platinum-based drugs, or immune checkpoint inhibitors—likely due to its lack of impact on intracellular drug concentrations or DNA damage checkpoint activity (122) (123) (124). Conversely, HCQ has been shown to exhibit synergistic antitumor effects when used in combination with inhibitors of mTOR (125–129), PI3K (43), EGFR (potentially) (130), and cell cycle-targeted agents (131). The underlying mechanism is attributed to HCQ-induced elevation of lysosomal pH, which subsequently activates pathways such as mTOR (132–135). Inhibition of the mTOR pathway can significantly enhance therapeutic outcomes. Furthermore, HCQ enhances the effectiveness of proliferation pathway inhibitors like sorafenib (136) and has been shown to improve survival rates in certain glioblastoma patients when administered alongside radiotherapy and carmustine (137). These results indicate that in the context of cancer adjuvant therapy, HCQ should be prioritized in conjunction with inhibitors that target pathways it influences, such as the mTOR pathway.

HCQ generally exhibits a favorable safety profile in oncology settings, often demonstrating manageable tolerability when used in conjunction with chemotherapy or targeted agents (138–143). However, the tolerable dosage of HCQ is contingent upon the specific combination regimen employed; it is typically lower in frontline chemotherapy combinations (144), with significant hematologic toxicity possible even at doses of 400 mg twice daily (145). Conversely, higher doses, such as 600 mg twice daily (140) or 800 mg/day (138), have been administered in combination with targeted agents, potentially reflecting an increased systemic burden associated with broad-spectrum anticancer drugs. Pharmacokinetic factors are also of paramount importance: HCQ may influence the absorption and exposure of co-administered drugs, such as by reducing the bioavailability of 2-deoxyglucose (146), increasing the exposure of MK-2206 (147), or heightening the risk of tamoxifen-associated retinopathy (148). Additionally, the absorption of HCQ itself may be adversely affected by proton pump inhibitors (149).

### 4.4 Other applications of hydroxychloroquine

Additional potential indications have been reported, including alopecia (150, 151), bacterial infections (152–154), oral erosive disorders (155–157), and osteoporosis (158, 159). Moreover, research indicates that HCQ may also confer beneficial effects in the management of gestational diabetes (160), hypertension during pregnancy (161), and specific bacterial infections occurring during pregnancy (162).

## 5 Outlook

HCQ, known for its antimalarial and immunomodulatory effects, acts as a “double-edged sword” due to its complex mechanisms, such as inhibiting TLR signaling and altering mitochondrial lipids, which offer therapeutic benefits but also pose safety risks. New strategies like chiral drugs and liposomal delivery aim to reduce toxicity, yet personalized medication models are essential. Future research should focus on: (1) Exploring HCQ’s interactions with G-quadruplex nucleic acids and mitochondrial proteins; (2) Examining synergy with immune checkpoint inhibitors; (3) Creating real-time toxicity monitoring. Understanding HCQ’s mechanisms will boost its clinical utility as a “multitarget star” drug.
